# Mechanism of Strain-Resistance Response of CNT/Polymer Composite Materials for Pavement Strain Self-Sensing Based on the Molecular Dynamics Simulation Method

**DOI:** 10.3390/polym17111427

**Published:** 2025-05-22

**Authors:** Xue Xin, Xingchi Zhao, Jing Gao, Zhanyong Yao, Yunzhen Li

**Affiliations:** 1 School of Civil Engineering and Architecture, University of Jinan, Jinan 250022, China; 18663969523@163.com; 2 School of Qilu Transportation, Shandong University, Jinan 250002, China; zxc19819366968@163.com (X.Z.); gaojing935@mail.sdu.edu.cn (J.G.); zhanyong-y@sdu.edu.cn (Z.Y.)

**Keywords:** smart road, in situ self-sensing, conductive sensitive material, strain-resistance response mechanism, molecular dynamics simulation

## Abstract

Embedded and real-time monitoring of pavement mechanical state changes based on the strain detected by self-sensing sensors of polymer/conductive composites is a new way for pavement health monitoring. Strain monitoring, using polymer-based composite mechanosensitive materials, requires the formation of effective conductive networks and conductive channels within the composite material so that the mechanosensitive material is electrically conductive at the macroscopic level. However, the deformation of the pavement structure is much smaller in magnitude, which is about hundreds or even tens of microstrains (10^−6^). Therefore, it is especially important to study the strain self-sensing mechanism of conductive composites at the με level. Micro- and nanostructured polymer composites have a complex structure with multiple layers, scales, and interactions, and thus present many difficulties when studying their microscopic conductive mechanisms. In this paper, the all-atom system of the micro-nanostructured composite mechanosensitive materials model was proposed with the help of molecular dynamics simulations. This achieved a breakthrough and realized the systematic study of the microscopic level of the relevant parameters of the composite’s conductivity from the molecular point of view to construct a relationship between the microscopic parameters, conductive network, and conductivity. The kinetic models of the micro-nanostructure and resin interface based on the molecular dynamics simulation technology were constructed to explore the dispersion state of the conductive filler, the interfacial interactions between the conductive filler and epoxy resin matrix, and the structural changes in the conductive network within the system under the tension state.

## 1. Introduction

Currently, the intelligence and smartness of roads mostly rely on roadside sensing devices (e.g., roadside cameras, radar) and mobile inspection vehicles to realize the sensing and guidance of vehicles and the external detection of road functional indicators. However, there is a lack of research on sensing means and the digitization of road structure ontology [1,2]. The current specification of the internal mechanical response of the road structure is an important indicator of road design, evaluation, and maintenance, while the actual construction and operation of the test parameters include bending subsidence, smoothness, etc. Lack of harmonization of monitoring indicators will result in an inconsistency between the design parameters of the pavement’s structure and the mechanical properties during the service period. The lack of an accurate basis for the design of a construction and maintenance program and other technical problems, which, in turn, lead to early structural damage, bring about an increase in the cost of maintenance, the deterioration of the road structure caused by safety accidents, and other social problems [3,4]. Autonomous and active perception of the mechanical state of the road structure can provide a basis for road construction, maintenance, and operation, and can also monitor and provide feedback on traffic information, thus promoting the construction of intelligent roads.

Embedded sensor monitoring is the core foundation for realizing active sensing of a pavement structure’s service conditions. With the development of advanced materials, a new technology of composites with self-sensing function shows good advantages for monitoring engineering structures [5,6], especially in solving technical difficulties, such as the poor deformation coordination of sensing elements and insufficient long-term working performance [7,8]. Composite materials show different changes in electrical and electromagnetic signals, etc., i.e., mechanosensitive properties, when subjected to different pressures, strains, temperatures, or other external factors [9,10]. Based on the excellent force-electric response properties, polymer-based composite mechanosensitive materials can be used for real-time monitoring of the mechanical properties of asphalt pavements [11,12]. However, due to the multilayer, multiscale, and multidimensional interactions of composite mechanosensitive materials, there is still much confusion about the mechanism of their strain-resistance response. In order to better reveal the strain-resistance response of self-aware composites with epoxy resin using a polymer matrix and various CNTs with different aspect ratios as the conductive fillers, the authors simulated the distribution, interaction, cluster aggregation, and contribution of conductive fillers, as well as the orientation change of a real polymer resin-cured crosslinking system and conductive carbon fillers by means of all-atom system molecular dynamics simulations and orientation changes of the conductive fillers.

### 1.1. Molecular Modeling Overview

**Figure 1:**
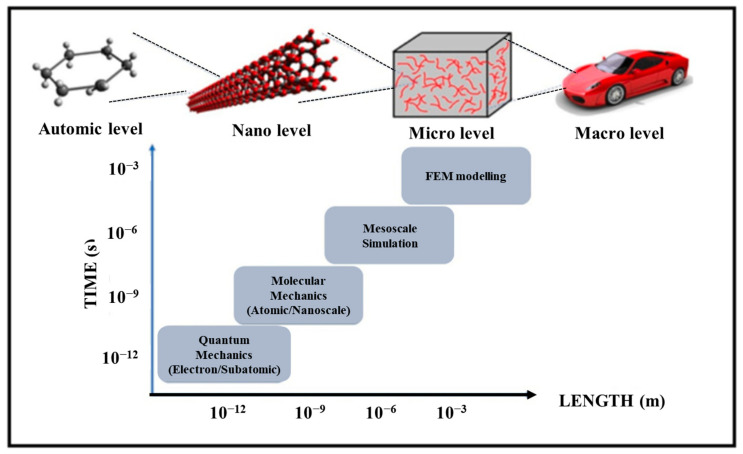
Time and length scale representation of simulation methods.

Simulation techniques in materials science offer novel ideas for exploring the possibility of combining different types of materials and predicting their properties. In recent decades, researchers have carried out in-system simulation studies of influencing factors, such as the scale, state of matter, and other conditions. Two types of simulation techniques commonly used in the study of material characterization are continuum-based and atom-based methods. Continuum-based simulation techniques are commonly used to study the properties of materials at both fine and macro scales, and one of the more commonly used is the finite element method (FEM) simulation based on meshing. Atom-based simulation methods mainly include molecular dynamics of tight bonding, Density Functional Theory (DFT), molecular dynamics (MD) simulations, and Monte Carlo (MC) simulations. Among them, the molecular dynamics of tight bonding and DFT are calculated using quantum mechanics based on the Schrödinger equation. For mesoscopic level calculations, on the other hand, MesoDyn and dissipative particle dynamics (DPD) approaches are usually followed, where MesoDyn was introduced in the 1990s based on the dynamic density flooding approach [13]. A summary of the simulation duration and simulation scale for each simulation technique is shown in Figure 1.

Molecular simulation mainly includes MC simulation and MD simulation. Among them, MD simulations are generally divided into three categories according to the system, such as the all-atom system, joint-atom system, and coarse-grained model; commonly used software includes Materials Studio (MS), LAMMPS, Nanoscale Molecular Dynamics (NAMD), etc. MD simulations utilize the classical Newtonian equations of motion, and the atoms are chosen as the smallest unit of action. Its study can be traced back to the 1950s, when it was first used to study the interactions between particles in a hard-sphere model consisting of hundreds of particles. This was followed by simulations of liquid systems and protein molecules in the 1960s and 1970s to explore their possibilities in different fields.

### 1.2. Molecular Simulation Study of the Electrical Conductivity of Composites

The two main types of simulation methods that have been used in the current research on the electrical conductivity of composites are MC- and coarse-grained-based molecular dynamics simulations.

**Figure 2:**
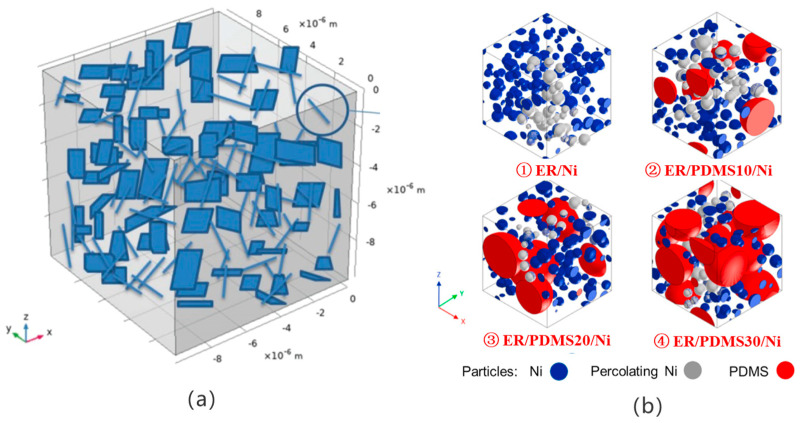
Composite model construction based on Monte Carlo simulation.

M. Haghgoo et al. [14] investigated the piezoresistivity and electrical conductivity of CNT/GNP polymer nanocomposites using the MC model. In the model, GNP and CNTs were simplified as randomly distributed thin rectangular cubes and cylinders, respectively, as shown in Figure 2a. After building the random CNT/GNP network, changes in electrical conductivity and relative resistance with strain were calculated. The results showed that the CNTs’ dispersion state and GNP size had a significant effect on the percolation threshold and the rate of change in resistivity with the strain of the nanocomposites. Zabiholah Zabihi et al. [15] investigated the percolation phenomena and electrical conductivity of GNP/poly (methyl-methacrylate) (PMMA) nanocomposites by using the MC method. Where the polymer composites were modeled as representative volume units (RVEs) with an edge length (l), and the GNPs were modeled as nanosheets with a thickness (t) and diameter (d), they were not permeable to each other. Nanosheets of the same size are considered randomly dispersed within the RVEs. The effects of the cubic cell size, as well as the set value of the tunneling distance, GNP diameter, and orientation on the electrical conductivity of GNP/PMMA nanocomposites were investigated using the model. The simulation results show that the volume fraction of GNP required to model the conductivity decreases as the tunneling distance set point increases. The larger the GNP diameter, the lower the percolation threshold and the lower the conductivity of the nanocomposites. GNP dispersed in a polymer with normal vectors parallel to the electric field has the lowest conductivity and the highest percolation threshold. Vinay Narayanunni [16] has developed a three-dimensional MC model to determine the critical volume fraction (CVF), conductive path length, and junction number of the composites. It was found that composites containing longer and smaller diameter fibers and polymer matrix particles resulted in lower CVF values. M. Jurča [17] used polymer blends with an optimized combination of epoxy resin (ER) and polydimethylsiloxane (PDMS) to reduce the percolation threshold (EPT) of Ni from 7.9 vol % to 3.7 vol %, and improved the conductivity of the composites by 7 orders of magnitude, as shown in Figure 2b. Meanwhile, due to the addition of PDMS, the adhesion of the composites was improved by 20%, and the impact strength was improved by 75%. For electrical conductivity simulations, the Monte Carlo method was used to calculate the electrical conductivity of polymer composites by detecting the growth of a conductive network with the help of the Python (Version 3.10) scripting language, by examining the size of the aggregates using the aggregation number in the polymer composites. Audrey Gbaguidi [18] constructed a model of hybrid nanocomposites filled with ellipsoidal GNPs and cylindrical CNTs and developed a model based on 3D tunneling continuous percolation. Using MC simulations, the filler content, composition, and morphology were parametrically investigated to analyze the conditions required for the synergistic interaction of percolation and electrical conductivity. It was shown that for better dispersed fillers within the hybrid system, the electrical properties were related to the number of tunnel junctions per filler within the percolation network of the nanocomposites, and that hybrid composites filled with specific morphologies of GNPs and CNTs exhibited synergistic electrical properties. Zhu Xun [19] established spherical CB and GNP nanosheet models based on the study of the effect of particle size on electroseepage when a single particle is filled, and studied the nonlinear cooperative conductive effect of materials when mixed-filled. The results showed that the electrical conductivity of hybrid-filled composites with a specific morphology is more likely to exhibit a better synergistic effect when the diameter of the spherical particles is much smaller than the edge length of the sheet filler. From the above study, it can be seen that the simulation of electrical conductivity using 3D Monte Carlo geometrical modeling can be simplified for common conductive fillers into geometrical shapes, such as tubes, rods, or flakes, and the materials with relevant shapes can be assigned specified properties.

**Figure 3:**
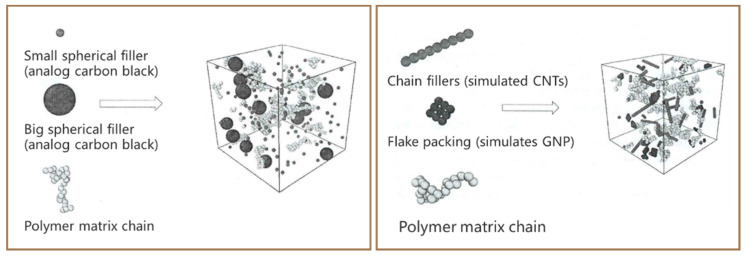
Composite model construction based on coarse-grained molecular dynamics.

In recent years, Beijing University of Chemical Technology has carried out much work in the study of electrical conductivity of composite materials using coarse-grained molecular dynamics [20,21,22,23,24,25,26], and the main simulation software used is LAMMPS. As shown in Figure 3, the process of simulating the electrical conductivity of composite materials with the help of coarse-grained molecular dynamics by LAMMPS is demonstrated. Several or some neighboring atoms in the molecular chain are regarded as “beads”; each polymer chain consists of dozens of beads. The dimensionless units of approximation m and σ are indicated as the mass and diameter of each bead. The conductive filler is also composed of “beads” by changing the diameter of individual beads to simulate the effect of the size of the conductive filler on the conductivity [27]. The effect of conductive filler’s shape on the conductivity and types of conductive filler for the synergistic or antagonistic effects is simulated by constructing chain beads to symbolize the CNTs and matrix beads to symbolize the GNP [22]. The interaction between polymer matrix and composite material is simulated by setting the degree of molecular chain modification or the degree of surface grafting of conductive filler [24].

However, it is well known that the coarse-grained model is not suitable for studying a particular polymer. Due to the limitation of the simulation scale, it is difficult to match the length of the polymer chain with the real polymer chain, and the conductive filler only focuses on the simulation of geometric morphology and specified properties, which does not reach the molecular scale. In 2021, Wang [21] combined the all-atom system with the coarse-grained model. In the polymer construction process, instead of directly adopting “beads” to simplify the construction, the all-atom system was used to crosslink the epoxy resins and curing agents with different functionalities according to the construction of the real molecular structure. Each molecule was then treated as a small ball for coarse-granulation, but the literature did not continue to explore the construction method of the composites in depth.

Comprehensive comparative research has revealed that the all-atom system simulation method has been studied in-depth when studying the mechanical properties [28], interfacial properties [3], and thermal conductivity [29] of composites. For example, N Nima et al. [30] studied the tensile properties of epoxy polymers using all-atom modeling and analyzed the structural changes in the resin during the tensile process. S. Faragi et al. [31] used molecular dynamics simulation to study the mechanical properties of epoxy resin reinforced with GNP and CNTs, and examined the effects of the doping amount of CNTs, the diameter of the CNTs and the amount of GNP on the mechanical properties of the composites, such as Young’s modulus, bulk modulus, and shear modulus. However, the electrical properties of composites are rarely studied by using the all-atom system. To better study the electrical conductivity of the constructed multilayered, multiscale, multi-interacting polymer composite mechanosensitive materials with complex micro-nanostructures and the changes in electrical properties under stretching, this paper will utilize the molecular dynamics simulation method to simulate the distribution, interaction, cluster aggregation, and orientation changes between the cured crosslinked system and the conductive carbon fillers of real polymer resins.

## 2. Molecular Dynamics Modeling of All-Atom Systems

### 2.1. Modeling

#### 2.1.1. Modeling of Polymer Matrix

**Figure 4:**
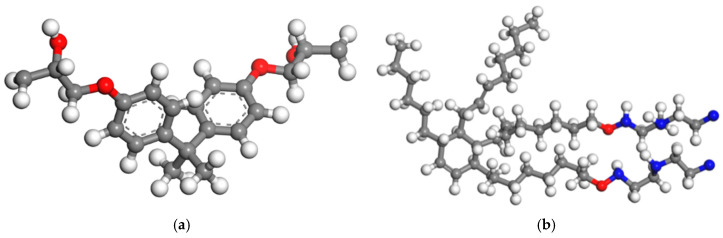
Reactive molecular configuration of epoxy resin and curing agent. (**a**) Molecular configuration of the epoxy resin (DGEBA), and (**b**) curing agent molecular configuration. Note: Black represents the C atoms, white represents the H atoms, red represents the O atoms, and blue represents the N atoms in the figure.

The epoxy resin and curing agent molecules were constructed using the Visualizer tool of Materials Studio software (B10VlA Materials Studio 2020 [20.1.0.2728], MS for short). The epoxy resin was selected as bisphenol A-type DGEBA, the polymerization degree of the molecule was set to 0, and the relative molecular mass was 340. The curing agent was selected as a low-molecular-weight polyamide curing agent, and the relative molecular mass was 730. The molecular monomers were optimized for further convergence to achieve the lowest energy by using the geometry optimization function in the Forcite module. Then, all the resin molecules and curing agent molecules were activated, and the epoxy bonds of the resin molecules were ring-opened and hydrogenated to present oxygen reactive sites. The amino groups (-NH_2_ -) in the curing agent molecules were deactivated by removing the active hydrogens to present nitrogen reactive sites. The structural morphology of the activated reactive state molecules is shown in Figure 4. Subsequently, the epoxy resin molecular monomer and the curing agent molecular monomer were again geometrically optimized for further optimized convergence, respectively, resulting in the lowest energy monomer conformation and a total charge number of 0 by the QEq method.

#### 2.1.2. Modeling of CNTs

**Figure 5:**
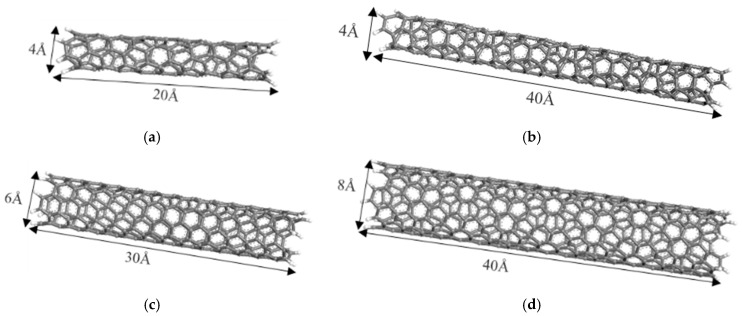
Molecular configuration of CNTs. (**a**) A1; (**b**) A2; (**c**) A3; (**d**) A4.

Four types of CNTs with different length-to-diameter ratios were constructed with the help of the nanotube structures in the MS library. The diameters of the CNTs were determined using the nanotube chiral indices N and M, and the lengths of the nanotubes were set using repeat units. Due to the unsaturated chemical bonding of the carbon atoms at both ends of the CNTs, it was necessary to add hydrogen atoms to the unsaturated carbon atoms for saturation. The molecular configurations of four CNTs with different length-to-diameter ratios are shown in Figure 5.

#### 2.1.3. Initial Modeling of Composite Mechanosensitive Materials

**Figure 6:**
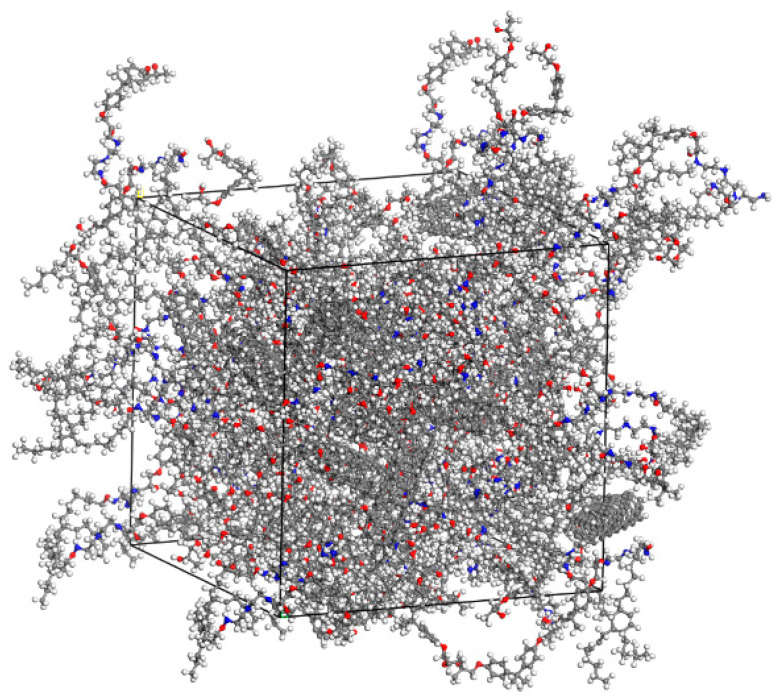
Cell model of CNT/ epoxy resin composite.

Using the Amorphous Cell Calculation module, 3D cell models containing 100 epoxy resin monomers, 25 curing agent monomers, and different types/quantities of conductive carbon fillers in a stabilized conformation were constructed. The initial cell model of conductive carbon filler/epoxy resin composite smart material with different mixing ratios was obtained, as shown in Figure 6.

### 2.2. Curing and Crosslinking Process

The curing crosslinking reaction of the system was simulated according to the reaction process between the bisphenol A-type DEGBA resin and the low-molecular-weight polyamide curing agent molecules. Each DEGBA molecule contains two epoxy functional groups, and each low-molecular-weight polyamide curing agent contains two primary and four secondary amine functional groups on average. To simplify the crosslinking process, the primary amine groups with higher reactivity are prioritized for crosslinking, followed by the secondary amine groups.

In the curing crosslinking process, according to the principle of “nearest neighbor,” to determine the number of active sites crosslinking, the “reaction cut-off distance” was set to control whether the curing reaction occurs. The reaction carbon atom on the bisphenol A-type DGEBA resin molecular fragment was arbitrarily searched and selected for the surrounding reaction nitrogen atom on the polyamide curing agent molecular fragment in the vicinity of the reaction nitrogen atom. If the reaction carbon atom–reaction nitrogen atom pair is within the set “reaction cut-off distance”, then the addition reaction between the reaction carbon atom and the reaction nitrogen atom will occur to form a chemical bond. Subsequently, the search distance is gradually increased until no new pairs of reacting atoms are within the reaction cut-off distance or the reaction has reached the preset crosslinking degree, at which time the model of the crosslinked system can be obtained. For the curing crosslinking reaction, the initial cut-off distance is set at 3.5 Å, and the maximum cut-off distance is set at 7.0 Å. The reaction step size is 0.5 Å, the predefined degree of crosslinking is 90%, and the temperature of the reaction is 433 K. The chemical bonds are established in such a way that the bonds do not pass through any carbon cycle structure. The process was carried out using the Perl language by writing the crosslinking script and embedding it in MS software, and the density after crosslinking was about 1.1 g/cm^3^.

**Figure 7:**
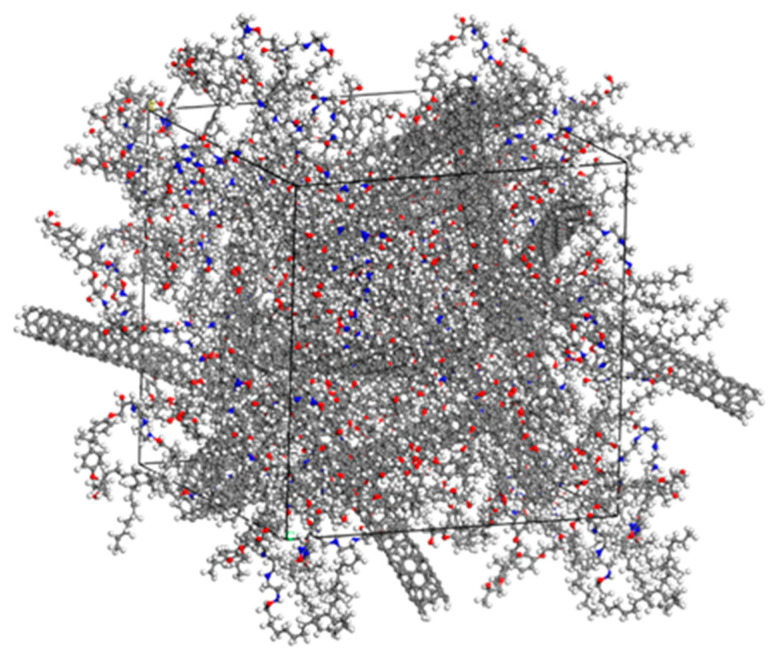
Cell model of the composite after curing the crosslinking reaction.

After the curing process, the cooling treatment of the composite system was simulated, with the temperature of the model gradually cooled from 433 K to 298 K with a cooling rate of 25 K/100 ps. At this time, the final model of the composite material was obtained. The cured, crosslinked, and cooled cell model is shown in Figure 7.

### 2.3. Molecular Force Field Selection

The molecular force field is the core of the molecular dynamics simulation. The molecular force field is a method of computationally fitting the various potential energies of each particle in a simulated system by taking the particle coordinates as the variables of the potential energy function, which can be regarded as an empirical expression of the potential energy surface. Compared with the quantum mechanical method, the computational time can be greatly saved in the calculation of the simulation system by a number of tens of thousands of particles. For common composite material systems, the total potential energy of the internal particles mainly includes three main categories of covalent bonding potential energy, coupling energy, and non-bonding potential energy, according to whether there is a chemical bond formation. The covalent bonding potential energy E_valence_ includes the stretching potential energy of bond length E_bond_, the bending energy of bond angle E_angle_, the torsion energy of dihedral angle E_torsion_, the out-of-plane bending energy E_oop,_ and the Urey–Bradley energy E_UB_. The coupling energy E_cross-term_ mainly includes the bond–bonding coupling energy E_bond-bond_, the angular–angular coupling energy E_angle-angle_, the bond–angular coupling energy E_bond-angle_, the angular–angular torsion coupling energy E_angle-angle-torsion_, the two-end bond–twisting coupling energy E_end_bond-angle_, and the middle bond–twisting coupling energy E_middle_bond-angle_. The non-bonding potential energy E_non-bond_ mainly consists of hydrogen bonding energy E_H-bond_, intermolecular interaction energy (van der Waals’ force energy) E_vdW,_ and electrostatic interaction energy (Coulomb force) E_columb_.

Currently, the commonly used molecular force fields in the molecular dynamics simulation process include the Universal, Condensed Mode Optimized Molecular Potential Field (COMPASS force field), cvff, Dreiding, pcff, etc. The potential energy function expressions of different molecular force fields are different, and the main difference lies in the non-bonding energy part. In this paper, the COMPASS force field is used to simulate the composite mechanosensitive materials to describe the intramolecular and intermolecular interactions. The COMPASS force field is also the most commonly used force field in MS simulation software to calculate the composite materials and small-molecule inorganic materials, and it can be used to describe the effective model when dealing with the mixing of two different systems with each other, which can not only improve the accuracy of simulations, but also improve the model calculation efficiency.

### 2.4. Geometric Optimization and Kinetic Equilibrium Process

In the geometry optimization process, the constructed composite model is geometrically optimized for a short period of time (5000 steps), with a step size of 1 fs, to obtain the stable composite configuration with the lowest energy. The four types of systems used in the molecular dynamics simulation process include the micro-regular system (NVE system), regular system (NVT system), isothermal isobaric system (NPT system), and isothermal enthalpy isobaric system (NPH system) [32]. In this paper, the system adopted in the kinetic equilibrium process is the NVT system in which the molecular dynamics equilibrium process is carried out at a constant volume V and 500 ps at 1 atm (0.0001 GPa). Fine precision is used throughout the simulation to improve the simulation accuracy. The temperature and pressure of the system were controlled by the Nose thermostat and Berendsen pressure controller, respectively, during the simulation process.

### 2.5. Dynamics of Motion Solving Equations

The cell in the MD simulation can be regarded as a system composed of several particles, and its solution process is to calculate the motion state of each particle at time t according to Newton’s laws of motion, and then solve the motion state after a time interval of tiny time Δ*t*. The equations of motion of a number of random particles can be combined to form a system of equations and then solved, and the positions and velocities of all the particles in the system at any time can be obtained by repeating the calculation many times. In this paper, the Velocity-Verlet algorithm is used to iteratively calculate the equations of motion. The Velocity-Verlet algorithm can calculate the position, velocity, and acceleration of the particles in the system at the same time with very high accuracy. The Velocity-Verlet algorithm’s specific position and velocity formulas are shown below.

(1) $$r_{i}\left(t+∆t\right)=r_{i}\left(t\right)+\frac{1}{2}{∆t}^{2}\frac{F_{i}(t)}{m_{i}}$$

(2) $$\nu_{i}\left(t+∆t\right)=\nu_{i}\left(t\right)+\frac{1}{2}[\frac{F_{i}\left(t\right)}{m_{i}}+\frac{F_{i}\left(t+∆t\right)}{m_{i}}]∆t$$

### 2.6. Characterization Methods for Conductive Properties

MD designs molecular dynamics simulations for all-atomic systems of the minimum order of atoms, which cannot be directly involved at the electronic level. Therefore, conductivity *Λ* is introduced into the simulation process to respond to the electrical conductivity of the composite mechanosensitive materials. Conductivity *Λ* represents the probability of the composition of a conductive network or conductive pathway, which varies from 0 ≤ *Λ* ≤ 1.

(3) $$Conductivity\;\Lambda =\frac{Number\;of\;Conducted\;Configurations}{Total\;number\;of\;configurations}$$

**Figure 8:**
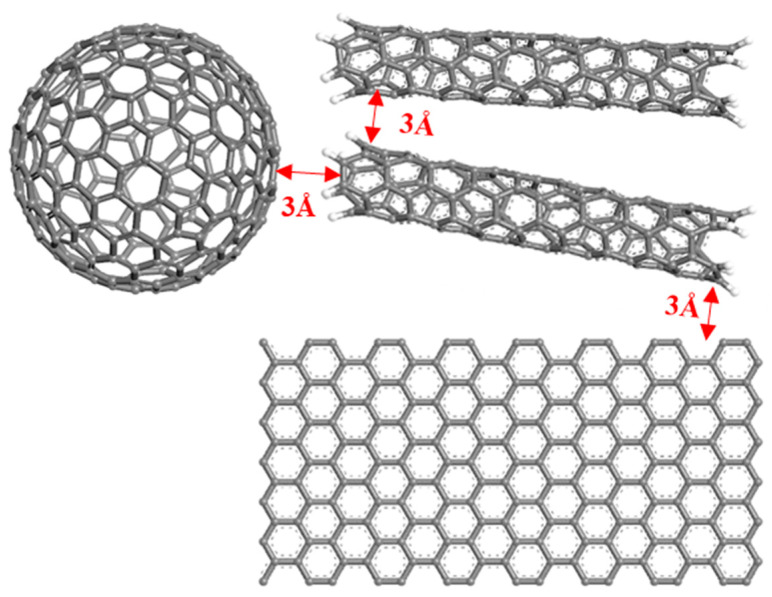
Diagram of tunnel distance TD.

When calculating the number of conductive configurations, a very important key point is how to determine that the conductive packing is conductive to each other. Therefore, tunneling distance (TD) is introduced, and if the gap distance between the surfaces of two conducting filler configurations is less than the TD, it is considered that the two conducting fillers are lapped successfully or conductive through the tunneling effect. According to the quantum chemical leapfrog theory [33], TD = 3Å is set, as shown in Figure 8. It should be noted that the magnitude of the conductivity Λ is only 1 order of magnitude, but the conductivity of the actual composites can change by 10–100 orders of magnitude near the percolation threshold, so the conductivity Λ is only used to qualitatively characterize the conductivity of the composites.

After the completion of crosslinking, geometry optimization, and kinetic equilibrium of the system, 5000 configurations of the system were statistically calculated to obtain the conductivity Λ with an output time interval of 0.01 ps for each configuration. The statistical calculation process was carried out by embedding the crosslinking script into the MS software after writing it in the Perl language. The rules for determining the conductivity of the cell system are as follows: at the initial stage, after the completion of the cell system, each conductive filler was assigned a corresponding consistent molecular number and cluster number from 1-N, with N being the total number of conductive fillers contained in the system. Whether any two conductive fillers are conductive or not is determined according to the set tunneling distance, TD. If they are conductive, the two conductive fillers are combined into one cluster, and the smaller of the two is selected for the named serial number. For each system configuration at a certain moment in the cell system, if there is a cluster that can penetrate the corresponding direction, the configuration is considered to be conductive at that moment. The conductivity of the system is obtained by counting the conductivity states of 5000 configurations of the system.

## 3. Conductive Network Analysis and a Conductivity Study in a Static State

### 3.1. Conductivity Λ in a Static State

**Figure 9:**
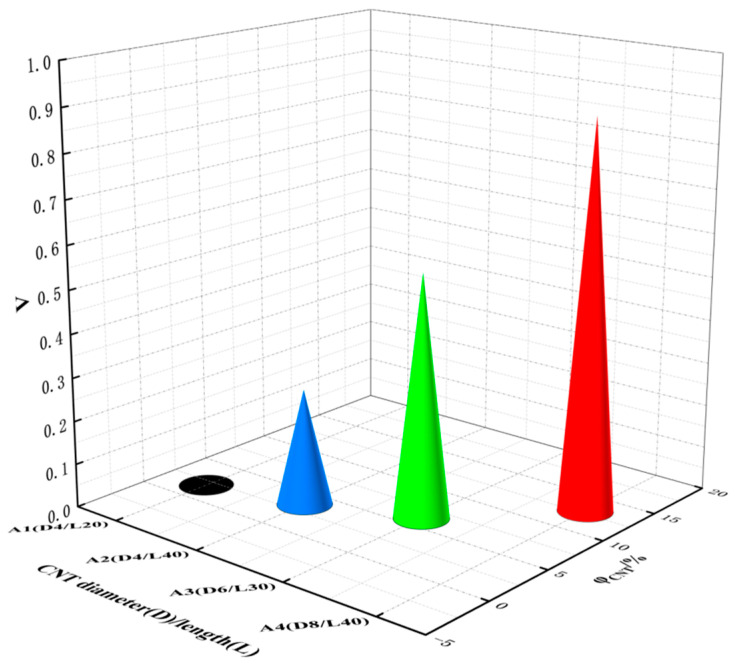
Conductivity Λ of composites with different CNT aspect ratios.

**Figure 10:**
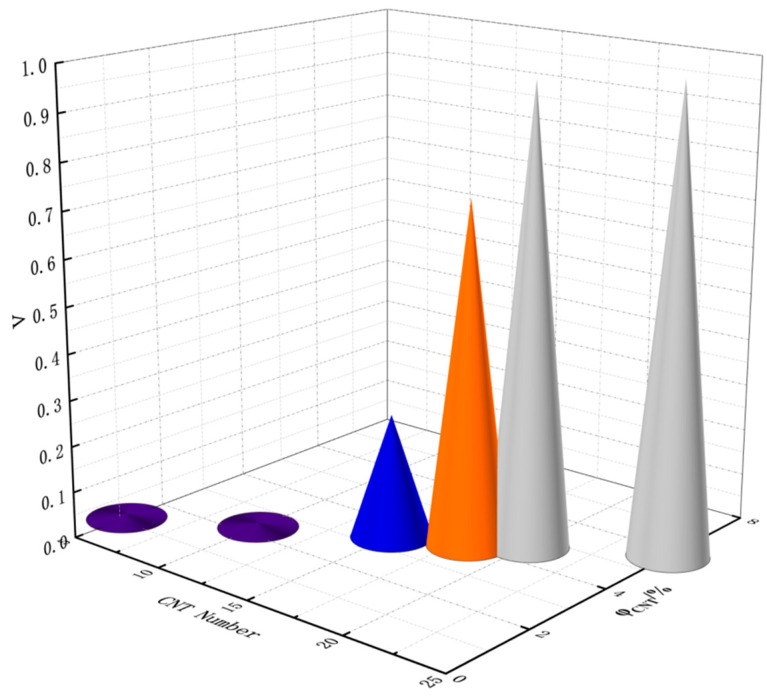
Conductivity Λ of composites with CNTs of different aspect ratios.

In order to study the effects of the type and doping of conductive fillers on the conductivity of pavement strain self-sensing materials, statistical studies were carried out on the conductivity Λ of two types of composites constructed with different aspect ratios of CNTs and different amounts of doped CNTs, respectively. The corresponding invariants were set under each system. Fixing the number of CNTs in the crystal cell system, the variation in the conductivity Λ of the four composite mechanosensitive materials with different aspect ratios of CNTs and volume fractions φ is shown in Figure 9 and Figure 10. For the composite system of CNT_A1 (diameter of 4 Å and length of 20 Å) with a volume fraction of 1.95%, the conductivity is nearly 0. As the diameter and length of the CN tubes increase, the conductive network is gradually formed, and the conductivity increases dramatically. With the same number of CNTs doped, the conductivity Λ of CNT_A4 (8 Å in diameter and 40 Å in length) reaches 0.90, which is far beyond the threshold of conductive percolation and is basically in a state of complete conductivity. In the molecular dynamics simulation of this study, after completion of crosslinking, geometry optimization, and kinetic equilibrium of the system, the data were counted for 5000 configurations of the system, with an output time interval of 0.01 ps for each configuration, and the integrated calculation of conductivity.

**Figure 11:**
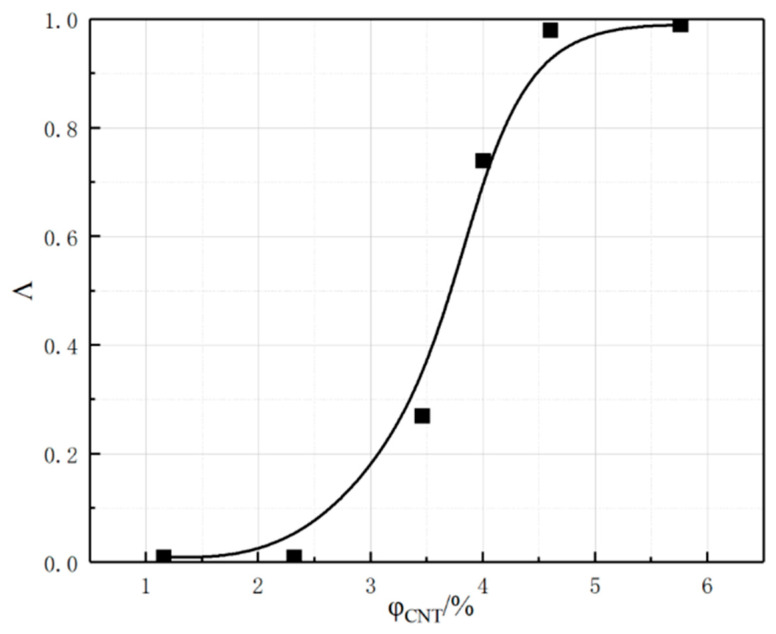
Conductivity Λ of composites with different CNT contents.

The CNTs_A2 with a diameter of 4 Å and a length of 40 Å were fixedly selected to investigate the effect of the number of CNTs (volume fraction) on the conductivity of pavement strain self-sensing materials, as shown in Figure 11, in which the CNT volume fractions φ were 1.16%, 2.32%, 3.46%, 4.07%, 4.60%, and 5.76%, respectively. When the CNT volume fraction φ is low, the conductivity of the composite system is very low and in the “insulating” state; however, with an increase in the CNT volume fraction φ, the conductivity Λ presents the “S-type” growth rule and then a sharp rise, and then tends to be stable. The sharp rise stage is the macroscopic manifestation of the conduction status near the conductive percolation threshold caused by the completion of the conductive network. When the volume fraction φ increases to 4.60%, the conductivity Λ tends to be close to 1, which indicates that the conductive network has been completely constructed. The conductive filler in the system can be completely through the whole composite material, and if the volume fraction of the conductive filler continues to be increased, there is basically no effect on the change in conductivity Λ.

### 3.2. Radial Distribution Function RDF of Conducting Fillers

The electrical conductivity of the composites is closely related to the degree of dispersion and dispersion state of the conductive filler in the polymer matrix. The effective dispersion state is crucial for improving the electrical conductivity and mechanical properties of pavement strain self-sensing composites. In this paper, the Radial Distribution Function (RDF) is introduced to quantitatively characterize the dispersion of different conductive filler systems. The density of filler particles around the conductive filler is characterized in MS by solving the ratio of the regional density and the global density of the particles in the periodic boundary box. The statistical formula for the RDF is:

(4) $$g_{AB}\left(r\right)=\frac{n_{AB}(r)}{4\pi r^{2}dr(N_{A}N_{B}/V)}$$

where *N_A_* and *N_B_* are the numbers of conductive filler A and conductive filler B, and *n_AB_ (r)* is the number of conductive filler A–conductive filler B found within the radius (*r* − *Δr/2*, *r + Δr/2*).

**Figure 12:**
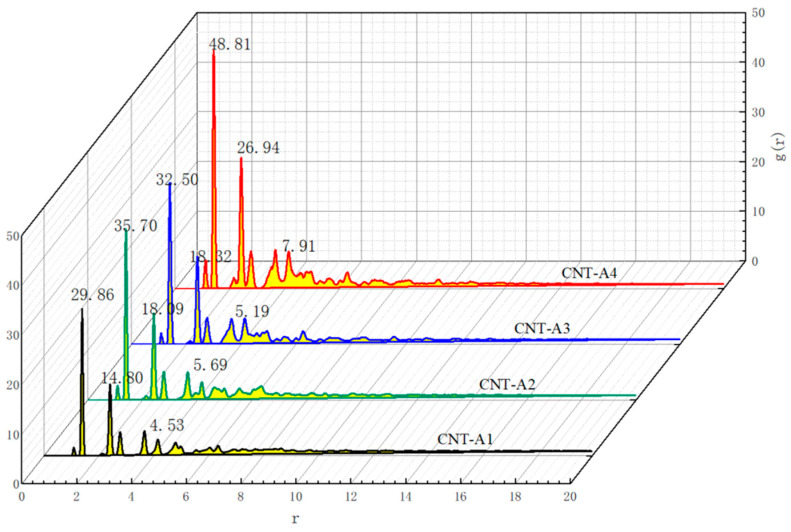
RDF of CNT–CNT in composites with CNTs of different aspect ratios.

**Figure 13:**
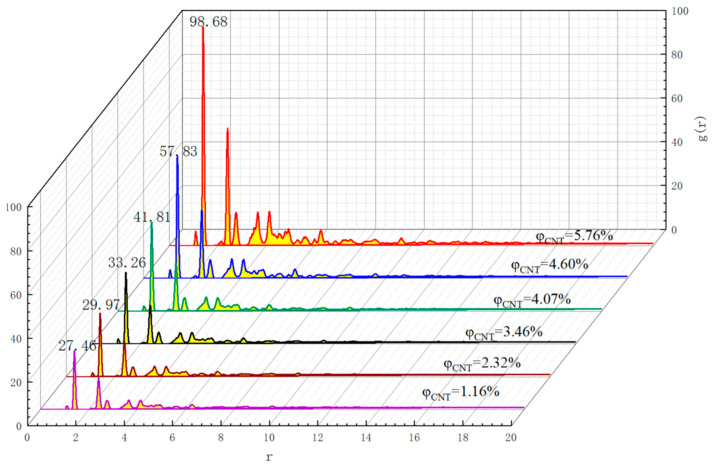
RDF of CNT–CNT in composites with different CNT contents.

Figure 12 and Figure 13 show the radial distribution functions *g(r)* of the conductive fillers in two types of polymer composite mechanosensitive materials with different CNTs’ length/diameter ratios and different CNT contents, respectively, where the truncation distances are set to 20 Å. It can be seen from the plots that the shapes of the radial distribution function curves of the different variables are basically the same for all types of systems and the positions of peaks in the *g(r)–r* curves are basically the same, which indicates that the system’s mixing and dispersion is consistent. However, the peaks in the radial distribution function are changed accordingly to the change in variables, and the larger peaks represent the greater density of filler at the corresponding r. From the figure, it can be seen that, in the cell model, CNTs show certain aggregation under the influence of van der Waals forces. But under the influence of the interaction between the polymer matrix and the conductive filler, the CNTs are not in direct contact with each other after the equilibrium state is reached in the model studied in this paper. With the increase in CNT doping, the peak is larger, indicating that the density of the conductive filler at the location is higher, and the aggregation is more obvious.

### 3.3. Maximum Cluster Size Cn and Total Number of Clusters N_c_

**Figure 14:**
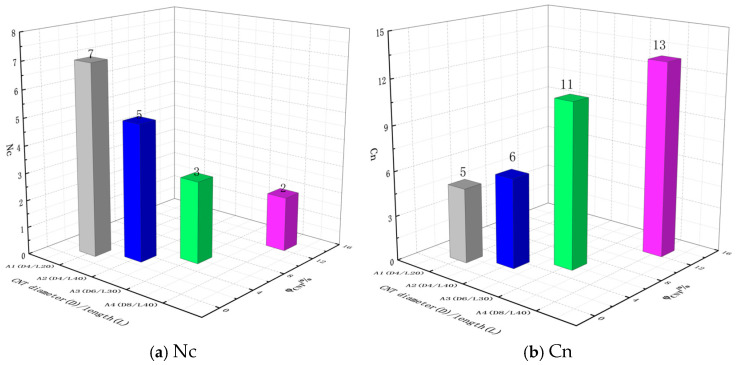
Nc and Cn of composites with CNTs of different aspect ratios.

In order to better characterize the conductive network of the pavement strain self-sensing materials, two parameters of maximum cluster size C_n_ and total cluster size N_c_ are further introduced in addition to the radial distribution function RDF. The maximum cluster size C_n_ characterizes the number of conductive fillers within the largest cluster. The C_n_ and N_c_ of four composites with different length-to-diameter ratios of CNTs are shown in Figure 14. The total number of clusters formed by the same CNTs varies greatly in the system. The shorter the diameter and length of CNTs, the larger the total number of clusters N_c_; and the smaller the maximal cluster size C_n_, the more difficult it is to construct a conductive network. In contrast, the number of clusters formed in the CNTs_A4 in the cell model is only three, in which the CNTs in the largest cluster account for 86.7% of the total CNTs in the cell, it means 86.7% of the CNTs in the system can be constructed to form a network structure and thus it is more likely to form a conductive pathway in the system, and the conductivity of the system is also higher.

**Figure 15:**
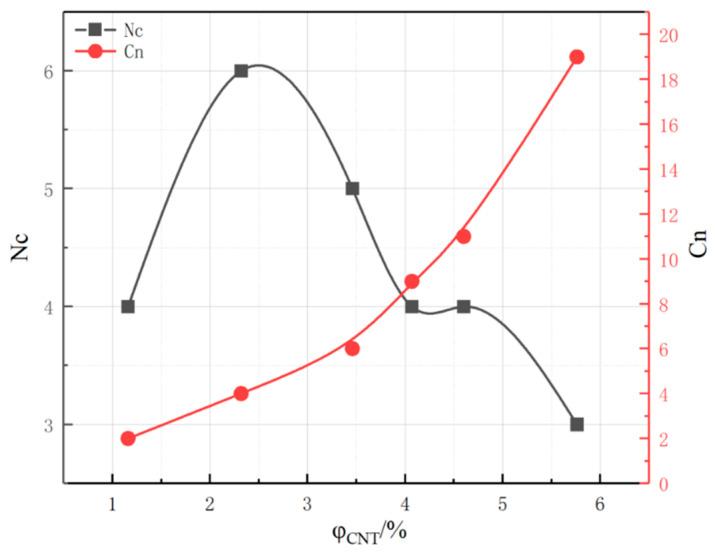
Cn and Nc of composites with different CNT contents.

With the type of CNTs fixed, the Cn and Nc of the system with an increasing CNT content are shown in Figure 15. With the increase in CNTs content, the maximum cluster size Cn grows larger and larger, indicating that the number of conductive fillers within the largest cluster constructed in the crystal cell system is increasing, and the volume of the formed conductive clusters grows larger and larger, which indicates that the conductivity is growing stronger and stronger. This study rule is consistent with the conductivity rule in Section 3.1, when the intracellular conductive cluster configuration exceeds the percolation threshold and has been fully conducted and then continues to increase the number of fillers within the conductive clusters, where the conductivity will not undergo a major change. The total number of clusters, Nc, shows a trend of increasing and then decreasing. Due to the low content of CNTs, the number of CNTs is very small and dispersed within the cell system, so the total number of clusters formed is higher. The total number of clusters Nc of CNTs at the lowest content (φ_CNT_ = 1.16%) is lower than that of φ_CNT_ = 2.32% Nc due to the small number of CNTs themselves.

### 3.4. Coordination Number CN

**Figure 16:**
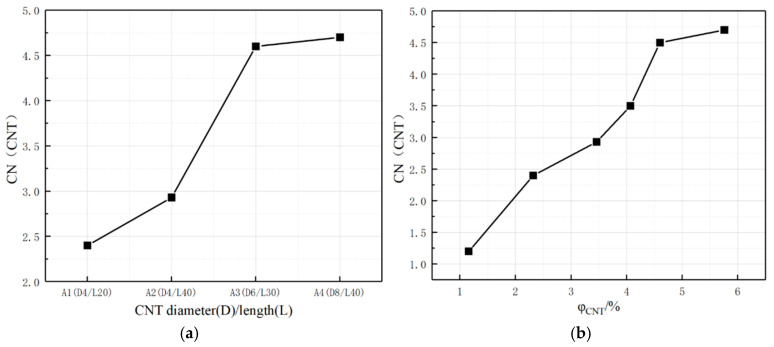
CN of conductive fillers in composites. (**a**) Different CNT length-to-diameter ratios; (**b**) Different CNT doping amounts.

A coordination number (CN) is further introduced to characterize the average number of other conductive fillers around a single conductive filler, which refers to the average number of CNTs around CNTs within a limited range in this paper. The coordination number statistics of the conductive fillers in the four systems are shown in Figure 16.

As shown in Figure 16, with the increase in CNTs length/diameter and the increase in CNTs content, the CNTs coordination number around the conductive filler CNTs gradually increases, indicating that the average distance between the CNTs within a limited range gradually decreases, which makes it easier to form a conductive network. Overall, the pattern presented by the coordination number CN is consistent with the pattern of the maximum cluster size Cn, but CN is a further quantitative statistical characterization of the degree of dispersion of the conductive filler within the composite system.

### 3.5. Distributional Probability P_N_

**Figure 17:**
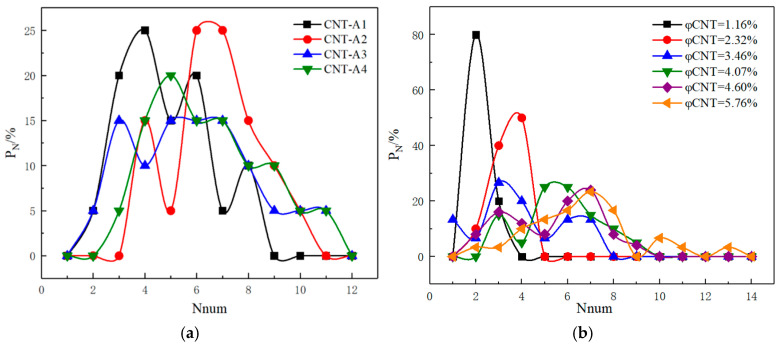
P_N_ of conductive fillers in composites with CNTs. (**a**) Different CNT length-to-diameter ratios; (**b**) Different CNT doping amounts.

In the distribution probability plot of pavement strain self-sensing materials, the horizontal coordinate N_num_ represents the number of CNTs present around the CNTs, and the vertical coordinate P_N_ represents the statistical distribution probability of the number of other fillers around the conductive carbon fillers, which can reflect the number of each filler connecting to the other fillers, and thus characterize the dispersion state between different types of fillers. The distribution probabilities under each curve sum to 100%. Figure 17 depicts the distribution probability of fillers in single conductive filler composites with only CNTs added. From Figure 17, we can see that the distribution probability of CNTs doped with the same number of CNTs but with different aspect ratios is more homogeneous with the increase in the length/diameter of the CNTs (e.g., CNTs_A3 and CNTs_A4). But, the CNTs with smaller lengths/diameters are more easily dispersed around a specific number of CNTs, which indicates that within a suitable length/diameter range, CNTs with larger lengths/diameters are more easily dispersed to form a good conductive network. However, the effective dispersion, curling, and winding of long CNTs should be considered in the actual production process. Only the CNTs with the shape of straight tubes were investigated in the simulation process of this present paper. For fixed types of CNTs, with the increase in the CNT’s volume fraction, the number of CNTs in the composite system also increases. Thus, under the premise of effective dispersion, the statistical distribution probability of CNTs connecting other CNTs around them also shows a tendency to move in the direction of increasing the number of CNTs.

### 3.6. Interaction Energy

Unlike the coarse-grained simulation system where the interaction coefficients between conductive fillers and polymers are set artificially, the MS with the all-atom system allows for the accurate calculation of polymer-various conductive filler interaction energies [34], and thus the effect of the interaction energies on the conductivity Λ and the dispersion state of the composites can be investigated. In the composite system, the interaction energy includes electrostatic, van der Waals forces, and non-bonding interactions in the molecular system. In the calculation process, groups were created for CNTs, conductive fillers, and resin matrices, respectively, and the interaction energy calculation script was written in the Perl language and then embedded into MS software for calculation. The corresponding interaction energy was displayed through the interface after the calculation is completed.

**Figure 18:**
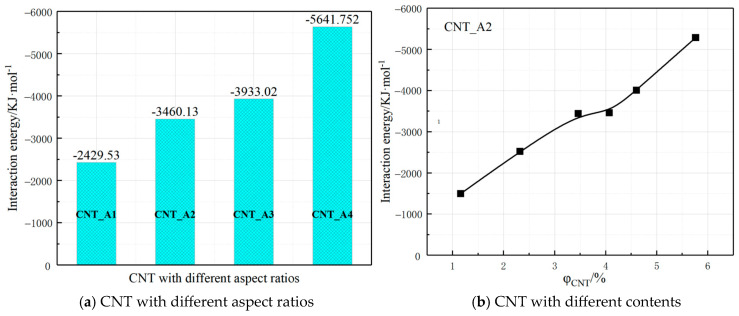
Interaction energy of CNT–epoxy in composites with CNT conductive filler.

The epoxy–CNT interaction energies in CNT-doped composites are shown in Figure 18. In agreement with the results of Jihua Gou [35] and Li Hao [28] et al. on epoxy resin/CNTs, the interaction energy of the polymer matrix-CNTs changes considerably with the structural changes of its different aspect ratios or the increase in the doping amount. The interaction energy of the same amount of CNTs_A4 with the polymer matrix is about two times that of CNTs_A1, and thus in the corresponding composites, CNTs_A4 is more likely to resist the van der Waals forces between the CNTs and be effectively dispersed by the interaction of the polymer matrix, which can be constructed to form a more optimal conductive network structure. In this paper, the interaction energy calculated statistically is the sum of the polymer matrix corresponding types of conductive fillers in the system, and with the increasing amount of CNT doping, the interaction energy between them and the polymer matrix also shows a basically linear growth trend.

## 4. Conducting Network Analysis and Conductivity Study Under Strain Conditions

The changes in the conductive network and conductivity of composite mechanosensitive materials under strain were further investigated to explain the mechanism of strain self-sensing for pavement based on composite mechanosensitive materials [20,36]. The composite system doped with CNTs_A2, with a volume fraction of 4.07% and an initial conductivity of 0.74, was selected to be stretched along the x-direction of the grain cell model, and the tensile strain was 50 με each time during the stretching process. The stretching process was equilibrated for 5 ps, and the system was subjected to a short-term NVT kinetic equilibrium of 5000 steps of 1 fs step length after each stretching. After the stretching process, the macroscopic conductivity Λ, the radial distribution function of the conductive filler inside the system RDF, the coordination number C_N_, and the probability of the conductive filler distribution P_N_ were analyzed before and after the stretching process, and the conductive filler orientation was characterized by combining it with the simulated snapshots of the composites during the stretching process.

### 4.1. Conductivity Λ Under Strain Conditions

**Figure 19:**
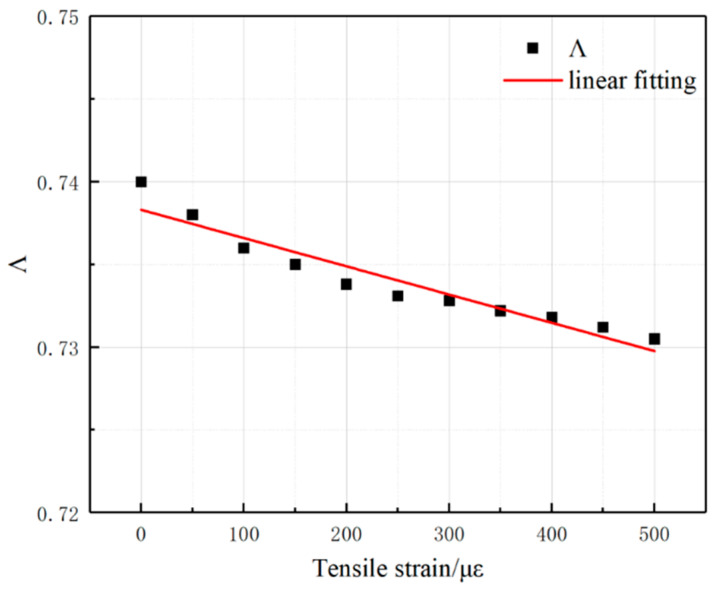
Conductivity Λ of composites during the stretching process.

At the end of each stretching, the conductivity of the equilibrium configuration for 5000 steps was counted and statistically calculated, focusing on the variation in the conductivity Λ of the composite mechanosensitive material during 10 stretches at 50 με /stretch and the cumulative stretching of 500 με, as shown in Figure 19. From the Figure 19, it can be seen that the conductivity Λ of the composite material shows a gradual decreasing trend with the gradual increase in tensile strain and is fitted to conform to the linear equation of Λ = 0.740 − 1.976 × 10^−5^ × με, which shows that the structure of the conductive network between the composites changes with the application of external transverse tensile force, leading to a change in the overall conductivity Λ of the composite material.

### 4.2. Structural Analysis of the Conductive Network Under Strain Action

**Figure 20:**
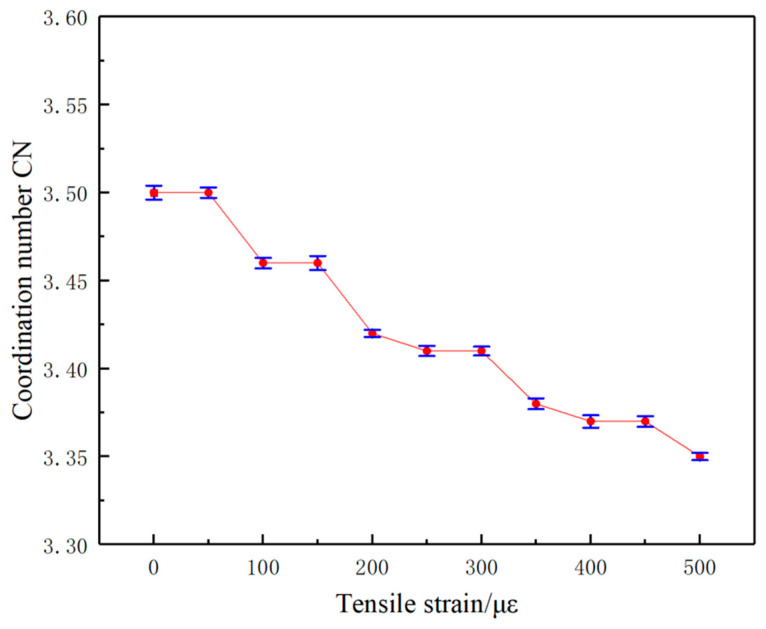
CN of conductive fillers in composites during the stretching process.

In order to investigate how the conductive network structure changes internally, this paper will further analyze the radial distribution function (RDF) of the conductive fillers under strain. Figure 20 lists the coordination number (CN) of the CNT conductive fillers in the composites during the stretching process. The coordination number (CN) characterizes the average number of other CNT conductive fillers around a single CNT conductive filler within the composites. With the gradual increase in tensile strain, the average number of coordinated CNTs around the CNTs gradually decreases from an average of 3.50 to 3.35, which indicates that the average distance between the CNTs has increased, leading to a change in the conductive network structure. However, the deformation of the internal conductive network structure of the composites is very small, as the tensile strain of the composites is only 500 με, which is the magnitude of change in the structural strain of our pavements.

**Figure 21:**
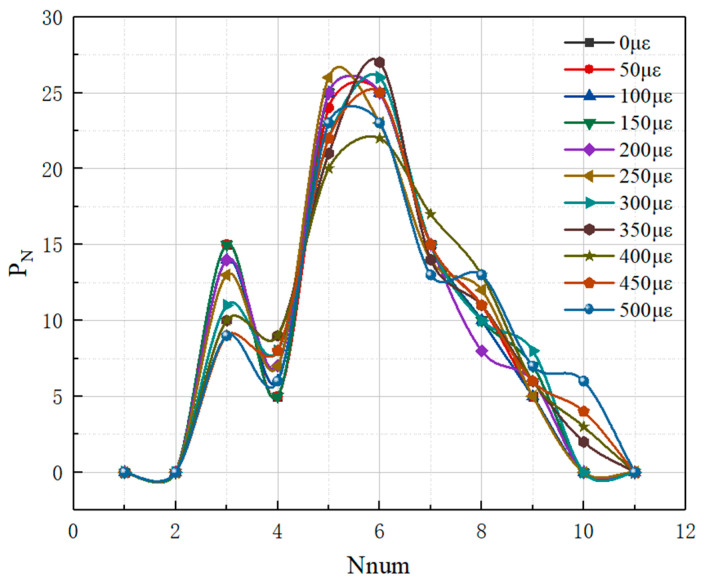
P_N_ of conductive fillers in composites during the stretching process.

In addition to the coordination number, the distribution probability (PN) of the conductive filler during the stretching of the composites was further calculated and analyzed to characterize the dispersion of the conductive filler, as shown in Figure 21. From Figure 21, it can be seen that with the gradual increase in tensile strain, the distribution probability of the conductive filler shows a gradual decrease in the peak value at a low number and a gradual increase in the peak value at a high number, which further indicates that the spacing between the conductive fillers in the system increases and the conductive fillers are more dispersed with the increase in the tensile strain, which leads to an increase in the system’s electrical resistance and a decrease in the conductivity Λ.

### 4.3. Conductive Filler Orientation Characterization

**Figure 22:**
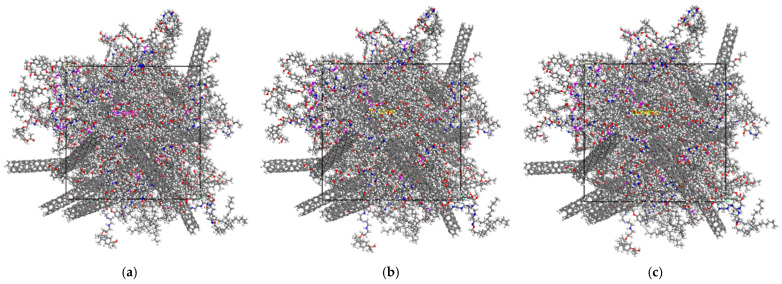
Simulated snapshot of the composite during the stretching process. (**a**) Unstretched; (**b**) Stretched with 200 με; (**c**) Stretching with 500 με.

In order to more intuitively represent the changes in the polymer matrix and conductive filler in the composites during the stretching process, the simulated snapshots of the composites from unstretched to stretched 500 με are listed, as shown in Figure 22. As can be seen from the positional changes in the O atoms (in red) and N atoms (in blue) combined in Figure 22, the molecular configuration of the matrix polymer of the composites shifted during the stretching process along the X-axis direction, which, in turn, caused a change in the electrical conductivity of the composites. Under the action of stretching, the change in the electrical conductivity of the composite material mainly comes from two factors. Firstly, the molecular chain segments of the polymer matrix epoxy resin are gradually straightened or the molecular chain segments move under the action of the external tensile force, and the degree of its loss will transfer the stress to the internal conductive network structure of the composite material. Secondly, the tensile stress makes the conductive filler in the composite material move or even rotate, thus causing the destruction and reorganization of the conductive network. The movement of tubular CNTs in the composites will be more obvious than that of spherical CB and bulky GNP.

**Figure 23:**
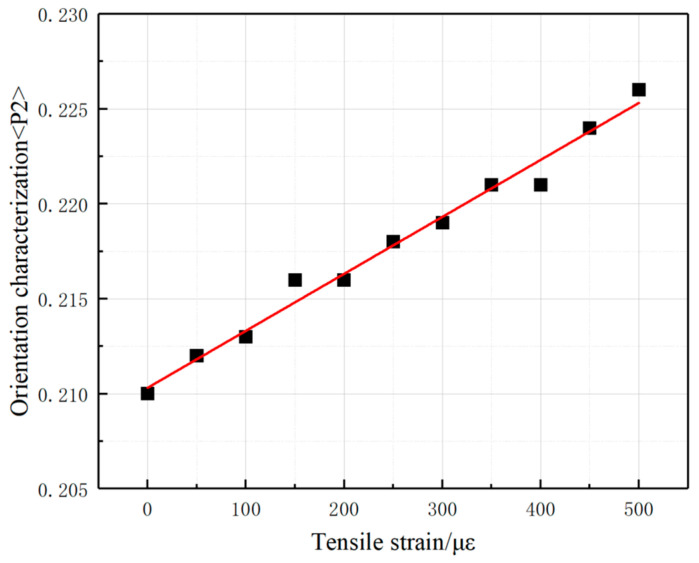
<P2> of CNTs in the composite during the stretching process.

In the absence of deformation, the orientation of conductive filler particles is isotropic. However, during the deformation process, how does the orientation of the nano-conductive filler change under the combined influence of external forces and internal interactions, thus causing a change in the electrical conductivity? In this paper, we continued to introduce the second-order Legendre polynomial *<P2>* to characterize the orientation of the conductive filler. Therefore, we further analyzed the composite system with CNTs as conductive fillers for the characterization of their orientation during stretching. The second-order Legendre polynomial <P2> is calculated as follows:

(5) $$<P_{2}>=\frac{3<{cos}^{2}\alpha >-1}{2}$$

where α is the angle between the straight line formed by the two end points of CNTs and the stretching direction (x-direction). If *<P2>* = 1.0, it means that the CNTs are parallel to the stretching direction; if *<P2>* = −0.5, it means that the CNTs are perpendicular to the stretching direction; if *<P2>* = 0, it means that the CNTs are randomly oriented. Figure 23 represents the variations in the average orientation of the CNT conductive fillers during the stretching process of the composites, and it can be seen that the value of the second-order Legendre polynomial *<P2>* of the CNTs in the composites is in the range of 0.21–0.23, which is between *<P2>* = 0 and *<P2>* = 1.0, indicating that the CNTs, as a whole, fall into random orientation and between parallel tensile directions. The second-order Legendre polynomial *<P2>* value of the CNTs increases with increasing tensile strain, indicating that the orientation of CNTs changes under external stretching and gradually approaches the parallel stretching direction.

## 5. Conclusions

This paper innovatively used the all-atom system of the molecular dynamics simulation method to construct a composite material model of micro-nanostructures to study the strain-resistance response for pavement strain self-sensing characteristics. In the all-atom system, the molecular model of epoxy resin and curing agent was constructed based on the polymer matrix, respectively, as well as the molecular model of CNTs with different aspect ratios. The COMPASS molecular force field was selected for model calculations, and the curing and crosslinking of polymer composites were emulated based on the crosslinking script written in the Perl language and embedded in MS software, combined with the actual chemical reaction process.

(1) Statistical studies on the conductivity Λ of the composites constructed with different aspect ratios of CNTs and different doping amounts of CNT systems in the static state were carried out, respectively, to analyze the effects of the aspect ratio and doping amount of CNTs on the conductivity Λ of the composites. The dispersion state of the conductive network inside the composites was also investigated quantitatively based on five parameters of conductive fillers’ radial distribution function (RDF), the maximum cluster size (Cn), the total number of clusters (N_c_), the coordination number (CN), and the distribution probability (PN). The interaction energy between the conductive filler and the polymer matrix was calculated, and the variations in the interaction energy with the parameters of the conductive filler were analyzed as well as the correlation relationship between the interaction energy, the conductivity and the dispersion state, which can reveal the interaction between the micro-, nanostructure, and resin interfacial behavior and conductivity behavior.
(2) Under the same doping amount, CNTs with a higher SSA present a greater number of CNTs and contact points, which can form a more perfect three-dimensional conductive network. However, dispersion, material price, and other issues should be considered at the same time, and we should not only focus on the high SSA of CNTs, but should comprehensively consider factors such as the preparation process, dispersion effect, sensitivity, and cost.
(3) The trend of the interaction energy is in perfect agreement with that of the parameters such as conductivity, maximum cluster size, and total number of clusters. It indicates the positive correlation between the interaction energy and the state of the conductive network, so the conductivity can be further improved by increasing the interaction energy.
(4) A typical composite system was selected to be stretched along the x-direction of the grain cell model with 50 με/step and a cumulative total of 10 steps. The changes in the macroscopic conductivity Λ, the radial distribution function (RDF) of the conductive filler, the coordination number (CN), and the probability of the distribution of the conductive filler (PN) of the composite material before and after stretching were analyzed. Thus, the conductive filler orientation was quantitatively characterized by combining with the simulated snapshots of the composite material in the stretching process. With the application of external transverse tensile force, the structure of the conductive network between the composites changed to some extent, the spacing between the conductive fillers in the system increased, and the conductive fillers were more dispersed, which caused the resistance of the system to increase and the conductivity Λ to decrease. From the molecular configuration change, the deformation of the molecular chain segments of the polymer matrix will gradually transfer stress to the conductive fillers, causing the destruction and reconstruction of the conductive network. Last but not least, the conductive polymer studied in this paper can be used as a core material for sensing in civil engineering deformation monitoring, body movement monitoring, traffic monitoring, etc.

## Data Availability

The original contributions presented in this study are included in the article. Further inquiries can be directed to the corresponding author.
